# Supplementary material for: Topologically frustrated dynamics of crowded charged macromolecules in charged hydrogels
Source: Nat Commun. 2018 Jun 8;9:2248. doi: 10.1038/s41467-018-04661-3 (PMC5993817; doi:10.1038/s41467-018-04661-3)
Supplement: Supplementary file 1 — Supplementary Information [file 41467_2018_4661_MOESM1_ESM.pdf]

Supplementary Information

Topologically Frustrated Dynamics of Crowded Charged Macromolecules in Charged Hydrogels

Jia & Muthukumar*

SUPPLEMENTARY FIGURE 1

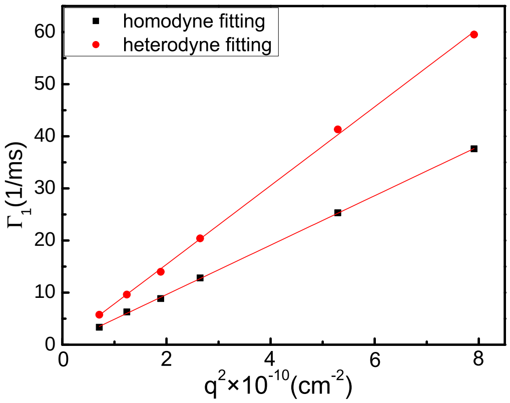

Supplementary Figure 1: Γ_1 as a function of q^2 of both homodyne and heterodyne fitting for the gel matrix.

SUPPLEMENTARY NOTE 1

From the slope of $\Gamma_1 - q^2$ obtained from both homodyne and heterodyne fitting, as shown in Supplementary Figure 1, we can get apparent elastic diffusion coefficient of the gel $D_A = (4.74 \pm 0.04) \times 10^{-7} cm^2/s$ from homodyne fitting and true elastic diffusion coefficient $D = (7.56 \pm 0.11) \times 10^{-7} cm^2/s$ from heterodyne fitting, respectively. Based on the relation $D_A = D/(2 - X)$, we get $X = 0.44$, which is the fraction of the intensity from the fluctuating component[1–8].

SUPPLEMENTARY FIGURE 2

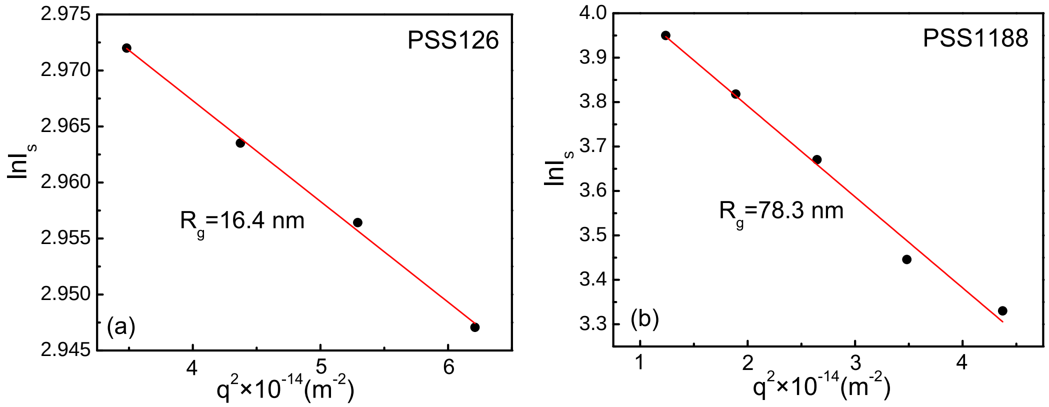

Supplementary Figure 2: Guinier plot for PSS in 0.1M NaCl dilute solutions. (a) PSS126 at 1mg/ml; (b) PSS1188 at 0.4mg/ml.

SUPPLEMENTARY NOTE 2

R_g of PSS with different molar masses in 0.1M NaCl solution (PSS concentration is below the overlap concentration) has been obtained by Guinier plot $I_s(q) = I_s(0)\exp(-q^2R_g^2/3)$. Supplementary Figure 2 shows two examples of the Guinier plot. The concentrations are 1mg/ml and 0.4mg/ml for PSS126 and PSS1188, respectively. From the slope of $\ln I_s - q^2$ plot, we get $R_g = 16.4$ nm and 78.3 nm for PSS126 and PSS1188, respectively.

SUPPLEMENTARY FIGURE 3

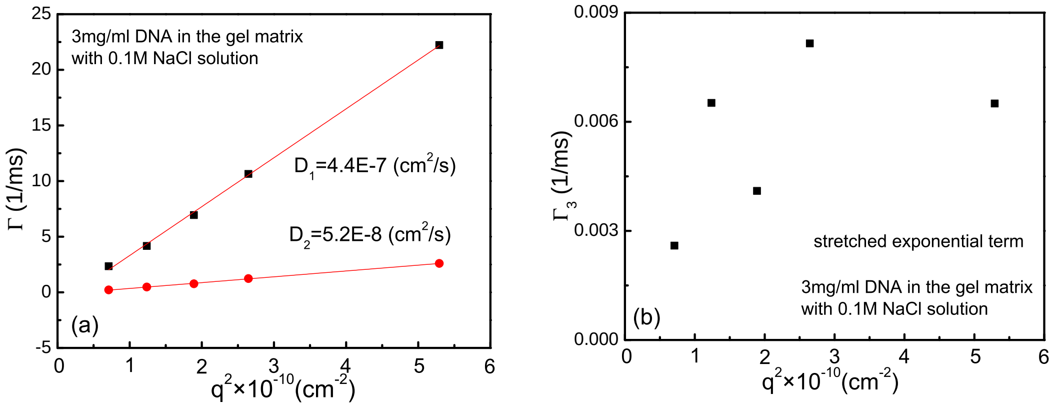

Supplementary Figure 3: (a) Relaxation rates of the first and second modes and (b) the third stretched exponential term as a function of q^2 for 3 mg/ml DNA in the gel matrix with 0.1M NaCl salt concentration. Both the first and second modes have a linear relationship so that from the slopes we can obtain the diffusion coefficient D_1 and D_2. The third mode does not have a linear relationship and it is non-diffusive.

SUPPLEMENTARY NOTE 3

The general formula for the electric field correlation function $g_1(t)$ is

$$g_1(t) = \int dm P(m) e^{-\Gamma_m t}, \tag{1}$$

where $P(m)$ is the probability of having m segments inside a mesh of size ξ and Γ_m is the rate of relaxation of m segments inside a mesh. From confinement free energy[9], $P(m)$ is given by

$$P(m) \sim e^{-A_\xi m}, \tag{2}$$

where A_ξ is a materials property depending on the mesh size and inter-segment interactions inside the mesh. The rate Γ_m depends on the local dynamics of the segments. If the local concentration inside the mesh is high enough, the hydrodynamic and the various excluded volume interactions are screened so that the rate follows the Rouse law[10],

$$\Gamma_m = \frac{A_R}{m^2}, \qquad \text{(Rouse)} \tag{3}$$

where A_R is a factor depending on the monomer friction coefficient and temperature. On the other hand, if the local concentration inside the mesh is low enough, the hydrodynamics and excluded volume interactions are not screened so that the rate follows the Zimm law[10] as,

$$\Gamma_m = \frac{A_Z}{m^{3\nu}}, \qquad \text{(Zimm)} \tag{4}$$

where ν is the size exponent defined through $R_g \sim M_w^\nu$, and A_Z is a prefactor depending on the segmental length and temperature.

Rouse dynamics. Substituting Eqs. (2) and (3) into Eq.(1) for the Rouse dynamics, we get

$$g_1(t) = \int dm e^{-A_\xi m - \frac{A_R}{m^2} t}. \tag{5}$$

This integral is evaluated using the saddle point approximation[11]. At the saddle point ($m = m^*$),

$$-A_\xi + \frac{2A_R}{(m^*)^3} t = 0, \tag{6}$$

so that

$$m^* = (\frac{2A_R}{A_\xi}t)^{1/3}. \tag{7}$$

Substituting this result for m in the exponent of Eq.(5), we obtain

$$-A_\xi m^* - \frac{A_R}{(m^*)^2}t = (\frac{27}{4}A_\xi^2 A_R t)^{1/3} \tag{8}$$

so that $g_1(t)$ is given by

$$g_1(t) \sim e^{-(\Gamma t)^{1/3}}, \tag{9}$$

where $\Gamma = (27/4)A_\xi^2 A_R$.

In general, for chains (even stiff chains) with the effective size exponent ν, and with hydrodynamic interaction fully screened, the characteristic time Γ_m is proportional to $m^{-(2\nu+1)}$,

$$\Gamma_m \sim \frac{1}{m^{(2\nu+1)}}. \tag{10}$$

Repeating the above derivation, the exponent of the stretched exponential becomes

$$\beta = \frac{1}{2\nu+2}. \tag{11}$$

For $\nu = 1/2$, this result is equivalent to Eq.(9).

Zimm dynamics. Substituting Eqs.(2) and (4) in Eq.(1), we get

$$g_1(t) = \int dm e^{-A_\xi m - \frac{A_Z}{m^{3\nu}}t}. \tag{12}$$

At the saddle point,

$$m^* = (\frac{3\nu A_Z t}{A_\xi})^{1/(3\nu+1)}. \tag{13}$$

Substituting this result in the argument of the integrand in Eq.(12), we obtain

$$g_1(t) \sim e^{-(\Gamma t)^{1/(3\nu+1)}}, \tag{14}$$

where $\Gamma = \frac{(3\nu+1)^{3\nu+1}}{(3\nu)^{3\nu}}A_\xi^{3\nu}A_Z$.

SUPPLEMENTARY FIGURE 4

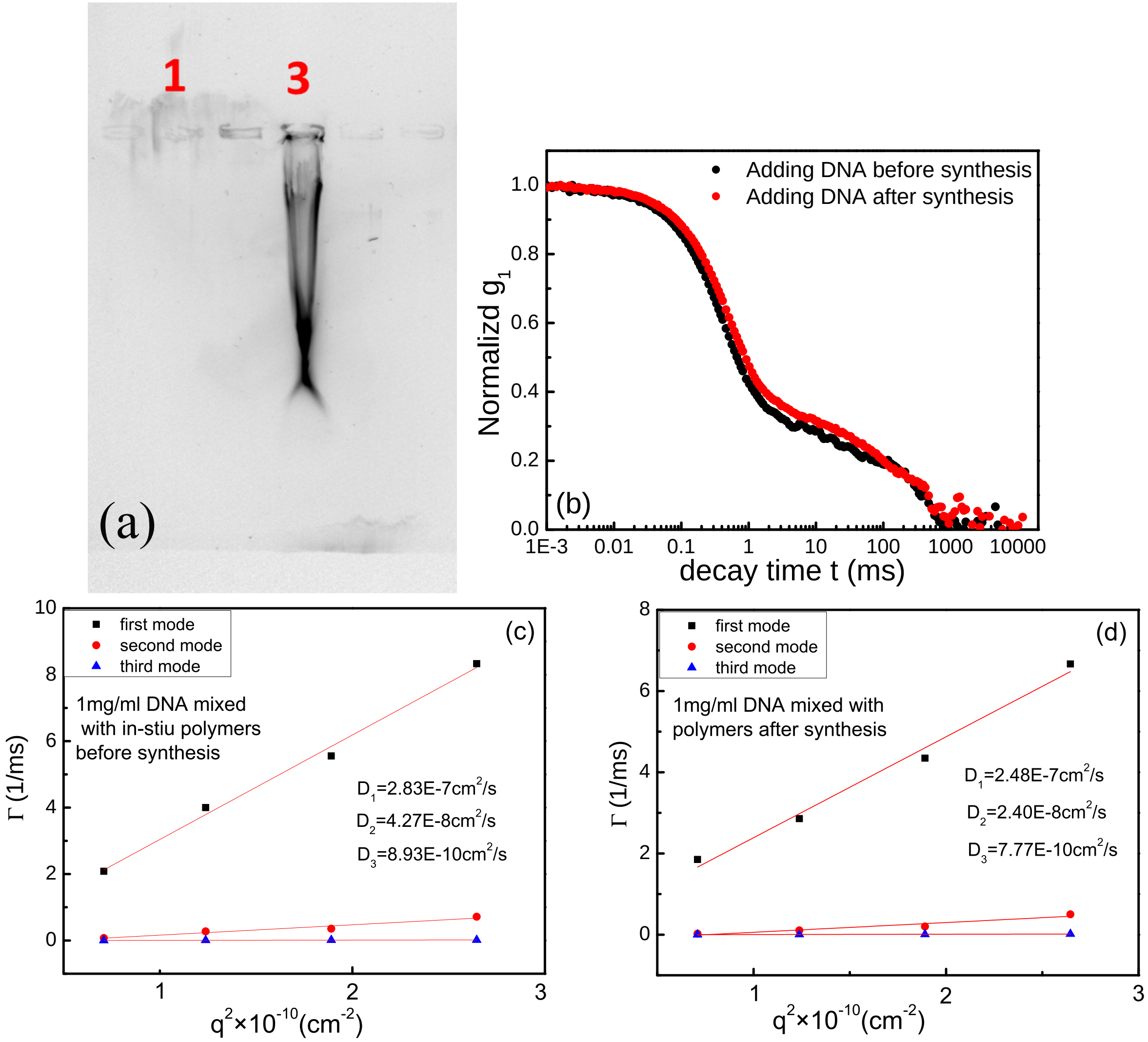

Supplementary Figure 4: (a) Gel electrophoresis of pieces of PAM-co-PAA gels without DNA (lane 1) and with 3 mg/mL DNA (lane 3) using the standard agarose gel with 0.1 M NaCl at 110 V for 1.5 hours. DNA escapes from the PAM-co-PAA gel and moves in the agarose gel under the electric field.(b) Field correlation function g_1 at the scattering angle of 30° for PAM-co-PAA solutions with 1mg/mL of DNA before and after the synthesis of the polymer, without cross-linker. DLS results for (c) PAM-co-PAA polymers mixed with DNA before synthesis; (d) PAM-co-PAA polymers mixed with DNA after synthesis.

SUPPLEMENTARY NOTE 4

In order to check whether the DNA has been chemically crosslinked to the in situ formed gel matrix or not, we conducted two experiments.

Experiment 1: We synthesized two samples of PAM-PAA hydrogels, one without DNA and the other with DNA, using the same experimental protocol described in the Methods

section. Small pieces of these samples were then subjected to gel electrophoresis in agarose gel with voltage of 110 V for 1.5 hours. The gel electrophoresis results are shown in Supplementary Fig.4a, where the reference lane 1 is for the PAM-PAA gel without DNA, and lane 3 is for the PAM-PAA gel with 3 mg/mL DNA. Under the electric field, PAM-PAA gel piece is stuck inside the well and cannot move, but if the gel piece contained DNA then the DNA would be released if it is not chemically cross-linked to the gel. It is evident from the result of lane 3 that DNA has escaped from the PAM-PAA gel matrix in the presence of an electric field, demonstrating that DNA is not covalently bonded to the PAM-PAA gel matrix.

Experiment 2: We synthesized two PAM-co-PAA solutions using the same protocol as in the Methods section, except now in the absence of the cross linking agent. In the absence of the cross-linker, the formation of PAM-co-PAA linear chains is still a free radical polymerization. The first sample is PAM-co-PAA polymers mixed with DNA during synthesis. The second sample was obtained by first synthesizing PAM-co-PAA polymers without DNA and after the reaction ended, the resultant polymer solution was mixed with DNA. The DLS data on these two samples are shown in Supplementary Fig.4c and 4d, respectively, where the DNA concentration is 1mg/mL for both samples. If DNA can form chemical bonds with the polymer chains by free radicals, an extra ultra-slow mode is expected to show up in DLS, which we did not observe. The field correlation function g_1 for these two samples at the scattering angle of 30^0 is given in Supplementary Fig.4b, showing that these are essentially identical. Besides, as seen from Supplementary Figs.4c and 4d, there are three diffusive modes for both cases and the diffusion coefficients are quite similar, independent of whether DNA was present or not during the free radical polymerization of the monomers. Therefore, DNA is not chemically cross-linked to the polymer chains in our system.

Supplementary References

1.Xue, J. Z.; Pine, D. J.; Milner, S. T.; Wu, X. l. Chaikin, P. M. Nonergodicity and light scattering from polymer gels. *Physical Review A*, **46**, 6550-6563 (1992).

2. Skouri, R.; Schosseler, F.; Munch, J. P. Candau, S. J. Swelling and Elastic Properties of Polyelectrolyte Gels. *Macromolecules* **28**, 197-210 (1995).

3. Shibayama, M.; Norisuye, T. Nomura, S. Cross-link Density Dependence of Spatial Inhomogeneities and Dynamic Fluctuations of Poly(N-isopropylacrylamide) Gels. *Macromolecules* **29**, 8746-8750 (1996).

4. Joosten, J. G. H.; McCarthy, J. L. Pusey, P. N. Dynamic and static light scattering by aqueous polyacrylamide gels. *Macromolecules* **24**, 6690-6699 (1991).

5. Fang, L. Brown, W. Dynamic light scattering by permanent gels: heterodyne and nonergodic medium methods of data evaluation. *Macromolecules* **25**, 6897-6903 (1992).

6. Geissler, E.; Hecht, A.-M.; Rochas, C.; Horkay, F. Basser, P. J. Light, Small Angle Neutron and X-Ray Scattering from Gels. *Macromolecular Symposia* **227**, 27-38 (2005).

7. Norisuye, T.; Tran-Cong-Miyata, Q. Shibayama, M. Dynamic Inhomogeneities in Polymer Gels Investigated by Dynamic Light Scattering. *Macromolecules* **37**, 2944-2953 (2004).

8. Pusey, P. N. Van Megen, W. Dynamic light scattering by non-ergodic media. *Physica A: Statistical Mechanics and its Applications* **157**, 705-741 (1989).

9. de Gennes, P-G. *Scaling Concepts in Polymer Physics* (Cornell Univ. Press, Ithaca, 1979).

10. Doi, M.; Edwards, S. F. *The Theory of Polymer Dynamics* (Clarendon Press, Oxford, 1986).

11. de Gennes, P-G. Relaxation anomalies in linear polymer melts, *Macromolecules* **35**, 3785-3786 (2002).
